# The Dysregulation Profile in middle childhood and adolescence across reporters: factor structure, measurement invariance, and links with self-harm and suicidal ideation

**DOI:** 10.1007/s00787-015-0745-x

**Published:** 2015-07-31

**Authors:** Marike H. F. Deutz, Sanne B. Geeraerts, Anneloes L. van Baar, Maja Deković, Peter Prinzie

**Affiliations:** Department of Child and Adolescent Studies, Utrecht University, P.O. Box 80.140, 3508 TC Utrecht, The Netherlands; Department of Pedagogical and Educational Sciences, Erasmus University Rotterdam, Rotterdam, The Netherlands

**Keywords:** Child Behavior Checklist, Dysregulation Profile, Comorbidity, Emotional dysregulation, Factor analysis, Suicidality, Developmental psychopathology, Measurement invariance

## Abstract

Recently, a phenotype of severe dysregulation, the Dysregulation Profile (DP), has been identified. DP consists of elevated scores on the Anxious/Depressed (AD), Aggressive Behavior (AGG) and Attention Problems (AP) scales of the Child Behavior Checklist (CBCL), Teacher Report Form (TRF), or Youth Self Report (YSR). A drawback in current research is that DP has been conceptualized and operationalized in different manners and research on the factor structure of DP is lacking. Therefore, we examined the factor structure of DP across multiple reporters, measurement invariance across gender, parents, and time, as well as links between DP and self-harm and suicidal ideation. Data from a large community sample were used (*N* = 697), covering middle childhood (*M*_age_ = 7.90, (SD = 1.16) and adolescence (*M*_age_ = 13.93, SD = 1.14). Mothers, fathers, teachers, and youth themselves reported on children’s emotional and behavioral problems using the CBCL, TRF, and YSR. Results indicated that in middle childhood and in adolescence, a bifactor model with a general factor of Dysregulation alongside three specific factors of AD, AGG, and AP fitted best, compared to a second-order or one-factor model. The model showed good fit for mother, father, teacher, and youth reports and showed invariance across gender, parents and time. Youth, mother, and father reported Dysregulation was uniquely and positively related to adolescent-reported self-harm and suicidal ideation. The DP is best conceptualized as a broad dysregulation syndrome, which exists over and above anxiety/depression, aggression, and attention problems as specific problems. The bifactor model of DP explains the uniqueness and interrelatedness of these behavioral problems and can help explaining shared and non-shared etiology factors. The exclusive link between the general dysregulation factor and adolescents’ self-harm and suicidal ideation further established the clinical relevance of the bifactor model.

## Introduction

Children with both emotional and behavioral problems show dysregulation across all three components of self-regulation: they have impairments in the ability to regulate affect (anxiety, depression), behavior (aggression), and cognition (attention problems) [1]. This phenotype of severe dysregulation is often represented by the Child Behavior Checklist Dysregulation Profile (CBCL-DP or DP [2]). DP consists of elevated scores on three syndrome scales of the Child Behavior Checklist: Anxious/Depressed (AD), Aggressive Behavior (AGG), and Attention Problems (AP) (or simply AAA-scales [2]).

The DP is not specific to the CBCL but an independent construct that has been found with a variety of questionnaires assessing emotional and behavioral problems, such as the equivalent questionnaire for teachers (Teacher Report Form; TRF, [3]) and the Strengths and Difficulties Questionnaire [4]. Consequently, we use the more general term of DP instead of CBCL-DP.

Research on the DP originated from research on childhood predictors of bipolar disorder. The CBCL—Juvenile Bipolar Disorder Profile (JBD) or Pediatric Bipolar Disorder Profile (PBD) were the original names for the profile consisting of elevated scores on the AAA-scales, as this profile was found to be predictive of bipolar disorder [5]. Several studies have since examined the link between DP or JBD/PBD and bipolar disorder and results have been inconsistent [6, 7]. Instead of a marker for later bipolar disorder, DP is now thought to identify children with poor regulation of emotion, attention, and behavior [2]. A growing line of research indicates that DP is a clinically relevant phenotype, uniquely predicting adverse outcomes like psychiatric problems [e.g., 1], pathological personality [8], and suicidality [e.g., 2, 9]. Research on the DP is also closely related to a small, but important field of clinical research that has demonstrated remarkably high rates of comorbidity between the clinical manifestations of the three components of self-regulation (affect: anxiety/depression; behavior: Oppositional Defiant Disorder (ODD); cognition: Attention Deficit Hyperactivity Disorder (ADHD) [10]).

Many of the studies that examined children with multiple types of psychopathology using the CBCL have focused on co-occurring internalizing and externalizing problems [e.g., 11, 12]. Attention Problems were not considered in these studies, as they are not part of the externalizing spectrum in the CBCL and TRF [13]. As Attention Problems especially are characterized by deficits in self-regulation [e.g., 14], which may be the core of comorbidity, the DP is especially relevant in research on comorbid behavior problems.

In a recent review, DP has been called “a useful index for identifying children and adolescents at risk for psychiatric problems” [15, p. 158). Given the widespread use of the CBCL in both research and practice, the possibility to identify relevant profiles on this measure to detect children who need early intervention in order to prevent aggravation of behavior problems into psychiatric problems is an important area of research [15].

## Different conceptualizations and operationalizations of DP

In the growing body of research on the DP, one major drawback is that DP is operationalized in different manners. These differences in operationalization make comparisons between studies difficult and hinder the progress of research on DP. How DP is operationalized relates to how it is conceptualized theoretically. Some researchers have claimed that DP might indicate a single syndrome consisting of diverse emotional and behavioral symptoms [e.g., 2]. This assumption is implicitly accepted by authors who used summed T-scores [e.g., 9, 16], latent class analysis (LCA) [e.g., 1, 8], or summed raw scores [e.g., 17] to define DP. Another view that has been proposed is of severe dysregulation as coexisting disorders, i.e., comorbidity [e.g., 18], which is implicitly accepted by authors who use a cut-off approach of summed T-scores per AAA-scale [e.g., 19] to define DP.

Another way to group different operationalizations of DP is into variable- or person-centered approaches. Where variable-centered approaches consider how variables are related to each other, person-centered analyses examine how these variables group within individuals. An example of a variable-centered approach is using a summed score for DP, while LCA is a person-centered approach. Factor analysis can be seen as a variable-centered approach. With factor analysis, a continuous underlying variable can explain the interrelations between AAA-scales. Although the mentioned studies have all contributed to the understanding of DP, research on the factor structure of DP is lacking. Examination of the factor structure can contribute to understanding the conceptualization of DP. Therefore, in this study, the factor structure of DP was examined by comparing three competing models. To determine the generalizability of the results, all models were examined separately for two different developmental periods, middle childhood and adolescence. In addition, the best-fitting model was examined for multiple reporters (mothers, fathers, teachers, and youth) and for measurement invariance across gender, parents, and time. External validity and clinical relevance were examined by assessing the relation of the best-fitting model for all reporters with self-harm and suicidal ideation as reported by the adolescents themselves.

## Factor structure of DP

**Fig. 1:**
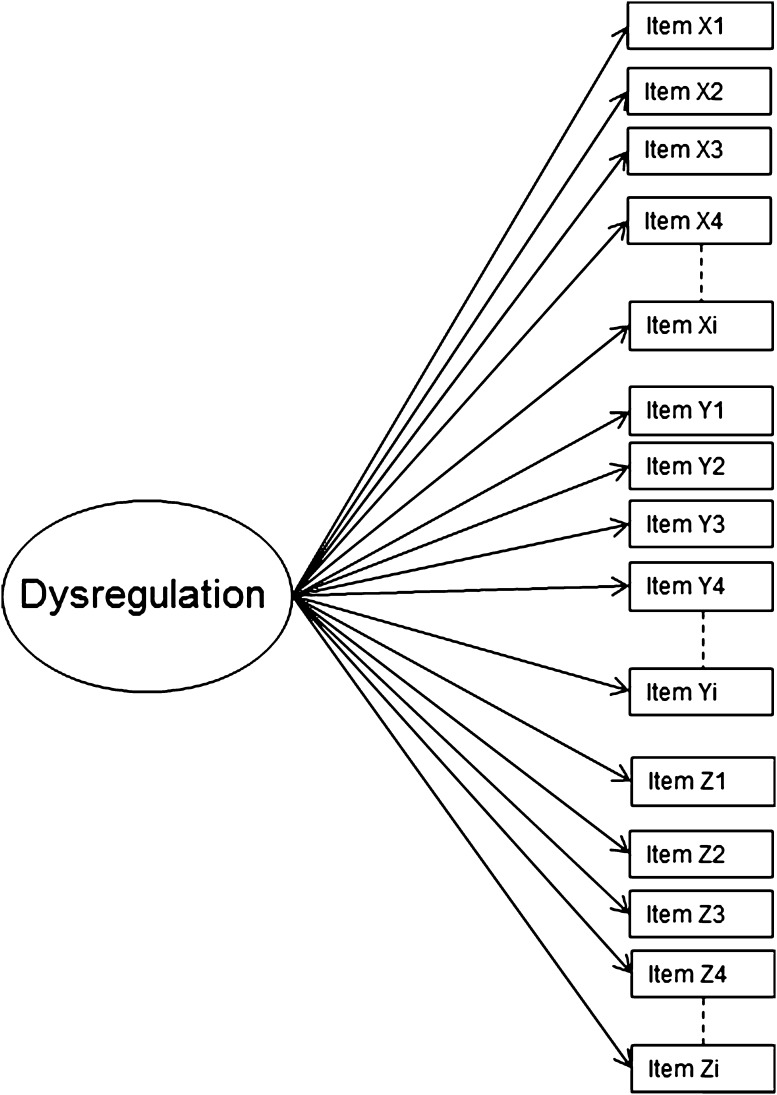
One-factor DP model

Although several studies have indicated that the constellation of the three AAA-scales into DP is meaningful, no actual research has used factor analysis to examine which structure best represents DP. Therefore, in this study, the factor structure of DP was examined by testing three competing models using Confirmatory Factor Analysis (CFA). The simplest model is the one-factor model (Fig. 1) in which the symptoms representing AD, AGG, and AP load onto one common factor of dysregulation. If this model would fit the data well, this suggests that the variance of these symptoms can be captured in one underlying factor, making it unnecessary to distinguish between AAA-scales. Therefore, this model fits well with the conceptualization of DP as representing a one-dimensional syndrome.

**Fig. 2:**
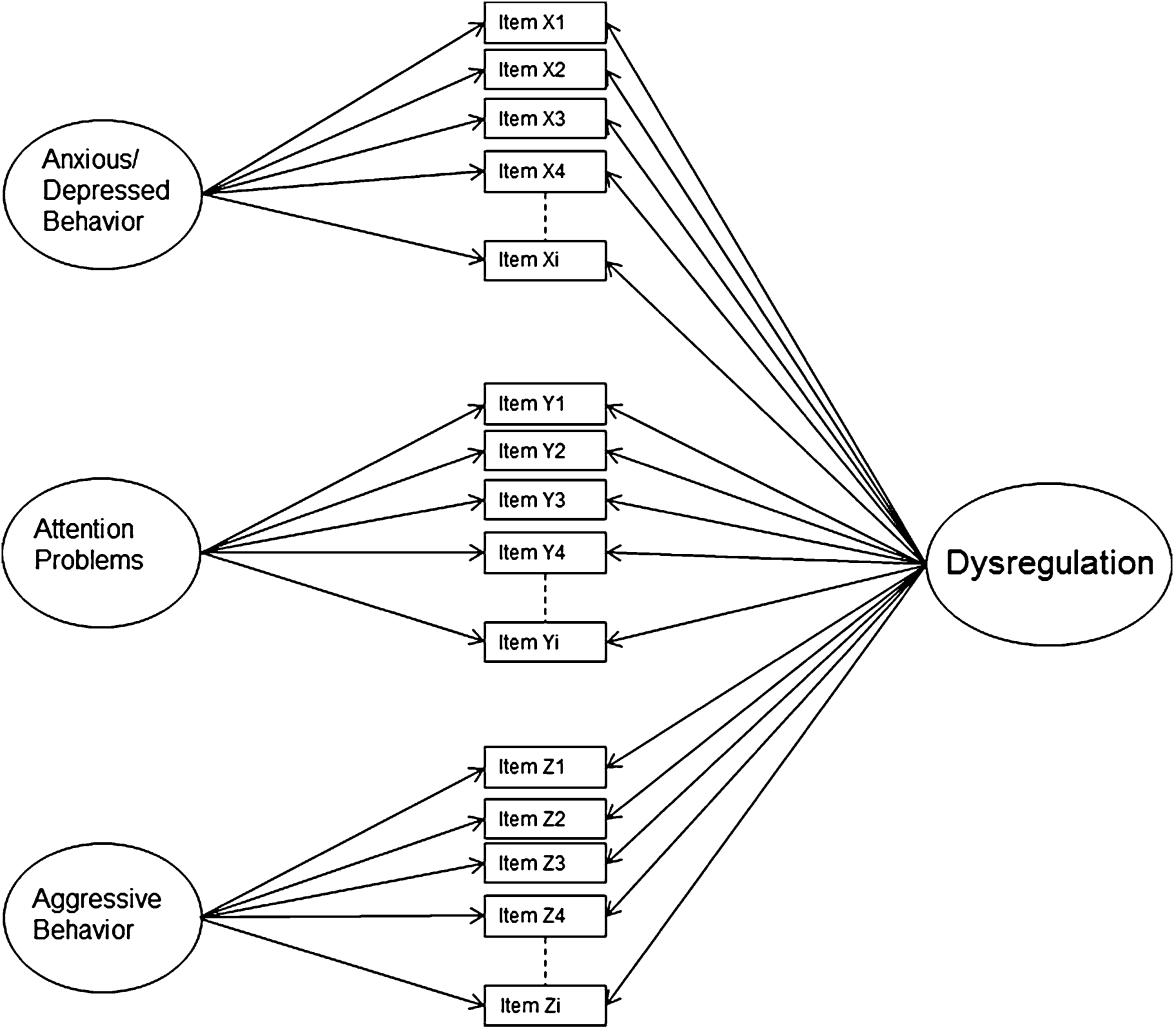
Bifactor DP model

Similar to a one-factor model, a bifactor model (Fig. 2), accounts for the hypothesis of DP representing a syndrome, as the emotional and behavioral symptoms comprising the AAA-scales share a general dysregulation factor. However, additional specific factors account for unique variance over and above the general factor. The bifactor model has been used in research on psychopathology before, for example, in research on ADHD [20]. It provides a new perspective on how to conceptualize psychopathology: next to disorder-specific factors, there might be a general underlying psychopathology factor (‘p factor’), which describes the individual liability towards developing psychopathology in general [e.g., 21, 22]. With regard to DP, the bifactor model implies a similar conceptualization: aside from distinct AAA-scales, accounted for by domain-specific factors, there is an underlying syndrome of dysregulation, represented by a general factor.

**Fig. 3:**
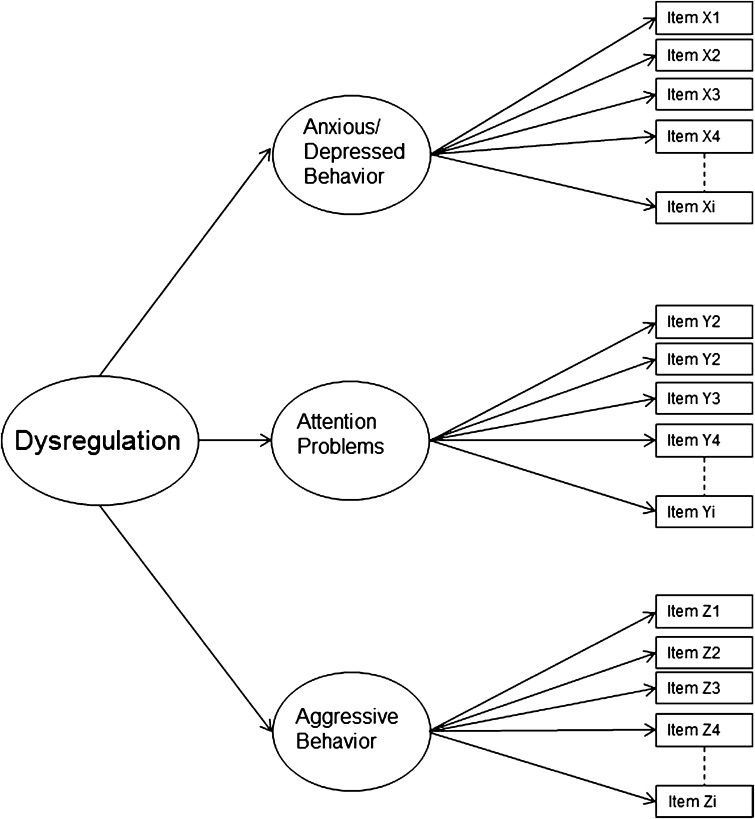
Second-order DP model

Lastly, a second-order model^1^ (Fig. 3) was tested that reflects three domain-specific latent factors representing the AAA-scales, which themselves load on a higher order factor of dysregulation. This model proposes that the three AAA-scales are distinct, but that an overarching dysregulation factor encompasses the interrelatedness, or comorbidity, between the three domain-specific factors. Therefore, second-order models are applicable from the perspective of DP as representing comorbidity.

The results of the test of three competing models have important clinical implications. Treatment guidelines could be differentiated according to the model that shows the best fit: a one-factor model would focus on a general treatment for Dysregulation, overlooking differentiation in the AAA symptoms present; a bifactor model would suggest that treatments of anxiety/depression, aggression, and attention problems could share identical components, most likely in targeting children’s self-regulatory capacities, whereas specific symptoms could be used for tailoring the treatment to the child’s needs; and a second-order model would suggest that Dysregulation could only be treated by taking into account the specific profile of symptoms shown in the three domain-specific latent factors.

Research on DP as well as research on the specific domains of anxiety/depression, attention problems, and aggression suggests that there are both shared and unique factors in the etiology of these forms of psychopathology [e.g., 10, 12, 23, 24]. This would indicate that a bifactor model would show the best fit, as this model allows for unique etiological factors to be examined for the specific and general factors separately. In a second-order model, this is not possible, as the relations between specific factors are thought to be entirely encompassed by one general factor. In addition, over a decade ago, it has already been suggested that covariation among symptoms of different psychiatric disorders cannot be explained by one general factor [10], indicating that a one-factor DP model would not show good fit. Therefore, it is expected that the bifactor model would show the best fit to the data.

## Measurement invariance across gender, parents, and time

When two groups are compared in terms of prevalence, means and correlates, it is typically assumed that both the measurement instrument and its underlying theoretical and psychological constructs are functioning in the same way across groups and time. However, these assumptions are rarely tested statistically [25]. In this study, we examined measurement invariance across gender, parents (as only father and mother reports are equivalent regarding the items constituting the AAA-scales), and time.

### Measurement invariance across gender

Previous studies examining DP showed inconsistent results concerning gender differences. Differences in prevalence were reported, with some studies indicating that boys were overrepresented [3, 19], whereas other studies indicated that DP was more prevalent for girls [26]. Another study showed no gender difference [9]. Before comparisons are to be made across gender, it is necessary to determine whether the factor structure of the construct of dysregulation is invariant (i.e., equivalent) across gender. Therefore, we tested measurement invariance for gender of the best-fitting DP factor model on mother reports (as these are most often used in clinical and research practice).

### Measurement invariance across parents

Many studies have examined (dis)concordance between mother, father, teacher, and youth reports of children’s emotional and behavioral problems using the Achenbach System of Empirically Based Assessment (ASEBA) [13], which consists of the CBCL, TRF, and YSR used in this study. These studies have shown that, in general, the concordance between reporters is low to moderate [27–29]. One study so far has reported on the cross-informant agreement on DP, using LCA to classify 6- to 18-year-old children. The DP latent class was identified across all informants (parents, teachers, and youth themselves). Cross-informant agreement was mild to fair, with Kappa’s ranging from 0.14 to 0.28 [3]. Although LCA (a person-centered approach) does not necessarily result in a similar conclusion when using factor analysis (a variable-centered approach), these results do inform on our hypothesis that the factor structure of DP is to be found for all reporters. The majority of studies on DP so far have used mother reports only or parental reports without differentiating between mother and father reports. In this study, the factor structure of DP was examined for father, teacher, and youth reports separately, in addition to mother-reports, to examine whether a DP structure could be found for all reporters. To further determine if the DP structure is similar across reporters, we examined measurement invariance for different reporters, fathers and mothers who answered the same item-set, with equivalent models. When measurement invariance between parents holds, the DP structure can be seen as equivalent for fathers and mothers.

### Measurement invariance across time

To examine whether DP is an equivalent construct in middle childhood and adolescence, measurement invariance across time was examined using mother reports. Results inform our knowledge of whether DP constitutes a similar construct in different developmental periods.

## External validity

Several studies have documented links between DP and parent- and self-reported self-harm and suicidal ideation, concurrently and over time, with the CBCL and other measures of suicidality [e.g., 1, 2, 9]. Given the robustness of this link, and the fact that suicidality is a form of severe adolescent psychopathology, we also examined the link between DP and suicidality to further establish the external validity of the factor model. We examined cross-informant associations of DP with youth-reported self-harm and suicidal ideation.

In sum, the factor structure of DP was examined in middle childhood and adolescence and across reporters. Measurement invariance was examined across gender, parents, and time. It was expected that a bifactor model of DP would best represent the data, indicating that DP consists of a general dysregulation factor as well as domain-specific factors for each of the AAA-scales. Based on a previous study in which a DP class across multiple reporters was identified [3], we expected that a similar factor structure would be present for mothers, fathers, teachers, and youth themselves, and that the structure would be invariant across parents. In addition, as this phenotype has been found for boys and girls separately [e.g., 30], we expected DP to be invariant across gender. Given the reported stability [23], we expected DP to be invariant across time. The dysregulation construct was expected to be positively related to adolescent-reported self-harm and suicidal ideation.

## Methods

### Procedure and participants

Data for this study were collected as part of the ongoing longitudinal Flemish Study on Parenting, Personality, and Development (FSPPD), a large community-based study. All parents provided informed written consent to participate in this study. As details of the recruitment procedures of FSPPD have been presented elsewhere [31], only the features of the methodology pertinent to the present article are presented.

For this study, we used data from the 2001 (middle childhood) and 2007 (adolescence) measurement wave to cover both middle childhood and adolescence as developmental periods. For middle childhood (*M*_age_ = 7.90 SD = 1.16), 49.8 % boys), data on the CBCL and TRF were available for 597 mothers, 560 fathers, and 697 teachers. In adolescence (*M*_age_ = 13.93, SD = 1.14, 47.4 % boys), data were available for 479 mothers, 445 fathers, and 414 teachers.

Mothers and fathers’ educational levels were as follows: elementary school (0.9, 3.0 %), secondary education (41.1, 43.3 %), non-university higher education (45.2, 34.4 %), and university (12.8, 19.2 %), respectively.

### Instruments

Mothers and fathers completed the CBCL/4-18 [32], teachers completed the TRF/4-18 [33], and adolescents completed the YSR/11-18 [34]. Parents and teachers were asked to rate 120 behavioral descriptions on whether they described the child now or within the past 6 months. Items were rated on a 3-point scale (0 = *not true*, 1 = *somewhat or sometimes true*, 2 = *very true or often true*).

For this study, items from the syndrome scales AD (CBCL: 14 items, TRF: 18 items, YSR: 16 items), AP (CBCL: 10 items, TRF: 20 items, YSR: 9 items), and AGG (CBCL: 20 items, TRF: 25 items, YSR: 19 items) were used. Cronbach’s αs for the AAA-scales ranged from 0.60 to 0.94 across reporters and time points (mean *α* = 0.83).

Item 45 (‘Nervous, highstrung, or tense’) was part of AD *and* AP in the 1991 versions of the CBCL, TRF, and YSR. However, in the revised versions from 2001 item 45 was considered part of AD only. As we wanted to avoid cross-loadings in our factor analyses, we considered item 45 to be part of AD only, consistent with the 2001 guidelines [13]. Items with generally less than 2.5 % endorsement across reporters and measurement waves were excluded from the analyses as these caused converging problems. The deleted items were items 80 (‘Stares blankly’) and item 97 (‘Threatens people’). A final number of items for the syndrome scales for parents, teachers, and youth, respectively, were 14, 18, and 16 for AD; 8, 18, and 8 for AP; and 19, 24, and 18 for AGG.

Trichotomous (0, 1, 2) responses for the items of the CBCL and TRF were dichotomized (0 vs. 1 and 2), consistent with other studies in which the factor structure of the CBCL was examined [e.g., 13, 35, 36].

To test the external validity of the DP, two items of the Youth Self Report (YSR) [34] assessing self-harm and suicidal ideation (thoughts or behaviors) were used. Item 18 asked ‘I deliberately try to hurt or kill myself’ and item 91 asked ‘I think about killing myself.’ A sum score was created of the two items, as both items were moderately correlated (*Spearman’s ρ* = 0.461). This sum score was again dichotomized. Data were available for 475 adolescents.

### Analyses

M*plus* version 7 [37] was used to perform the CFA. The Weighted Least Squares Means and Variances adjusted estimator (WLSMV) with delta parameterization was used to address categorical symptom ratings and resulting non-normality [38]. Model fit was evaluated using three primary indices: the comparative fit index (CFI), the Tucker–Lewis index (TLI), and the Root Mean Square Error of Approximation (RMSEA). Generally, values of CFI and TLI between 0.90 and 0.95 indicate acceptable fit, and values ≥0.95 indicate good fit. Values of RMSEA ≤0.08 indicate acceptable fit, and values ≤0.05 indicate good fit [39, 40]. Although Chi-Square is reported, it is not interpreted, as it is nearly always significant in larger samples and/or complex models [41]. When comparing models, Chi-Square difference tests for WLSMV estimator [see [37] were conducted, with significant Chi-Square values indicating a worse model fit.

To evaluate measurement invariance across gender, a multi-group approach was used in which metric invariance (shown by equivalent factor loadings) and scalar invariance (shown by equivalent intercepts) were tested. Following procedures described by Muthén and Muthén [37] for testing measurement invariance with categorical indicators using the WLSMV estimator and delta parameterization, two models were tested using mother reports. Model 1 was the less restrictive model in which thresholds and factor loadings were free across groups, scale factors were fixed at one in both groups, and factor means were fixed at zero in both groups. Model 2 was the more restrictive model in which thresholds and factor loadings were constrained to be equal across groups, scale factors were fixed at one in one group and free in the other, and factor means were fixed at zero in one group and free in the other. This model tested metric and scalar invariance, and when these conditions held, strong factor invariance could be assumed.

As a different constellation and number of items is used in teacher and youth reports (TRF, YSR) compared to parent reports (CBCL), measurement invariance across reporters could only be examined for mothers and fathers. Similar to testing measurement invariance of gender, two models were compared following procedures described by Muthén and Muthén [37] to examine measurement invariance across parents and across time. Due to the dependent nature of the data, a one-group model approach was used in which covariance of items and factors between parents or over time was specified [see 42].

As changes in CFI (ΔCFI) and RMSEA (ΔRMSEA) are the most widely used and empirically supported criterion to define invariance [39, 43], we used these indicators for measurement invariance across gender, parents, and time. Values of ΔCFI ≤ 0.01 and ΔRMSEA ≤ 0.015 indicate that the invariance hypothesis should not be rejected [43].

## Results

### Factor structure of DP

The three competing models on mother reports were compared for middle childhood and adolescence data separately. The least restricted model was the bifactor model, which consisted of three orthogonal first-order factors (AAA-scales), together with a general factor of Dysregulation on which all items of the AAA-scales loaded. This model was then restricted into a second-order model, which consisted of three first-order factors (the AAA-scales) loading onto one second-order Dysregulation factor. Subsequently, this second-order model was restricted into a one-factor model, consisting of all items of the AAA-scales loading only on one Dysregulation factor. Chi-Square difference tests were conducted to examine whether restrictions led to a significantly worse model fit. Table 1 presents the fit statistics of all three models in both developmental periods, as well as the results of the Chi-Square difference tests. As can be seen from this table, the bifactor model showed good fit in middle childhood and in adolescence. This fit significantly degraded when the model was restricted into a second-order model and subsequently into a one-factor model. Therefore, the bifactor model was selected for further analyses.

**Table 1:** Fit indices for the one-factor, second-order, and bifactor models of DP for middle childhood and adolescence using CBCL mother reports

Model	*χ* ^2^	df	RMSEA	RMSEA 90 % CI	CFI	TLI	Δ*χ* ^2^
Middle childhood							
1. Bifactor model	1281.294	777	0.033	[0.030–0.036]	0.938	0.932	
2. Second-order model	1496.990	816	0.037	[0.034–0.040]	0.917	0.912	2 vs. 1 (39) = 202.312, *p* < 0.001
3. One-factor model	2039.855	819	0.050	[0.047–0.053]	0.851	0.843	3 vs. 2 (3) = 198.549, *p* < 0.001
Adolescence							
1. Bifactor model	1142.179	777	0.031	[0.027–0.035]	0.952	0.947	
2. Second-order model	1321.710	816	0.036	[0.032–0.039]	0.934	0.930	2 vs. 1 (39) = 183.810, *p* < 0.001
3. One-factor model	1645.471	819	0.046	[0.043–0.049]	0.891	0.886	3 vs. 2 (3) = 170.506, *p* < 0.001

### Comparison of reporters

To examine whether the same factor structure holds across reporters, we replicated the bifactor model on father, teacher, and youth data. The fit indices of these tests are reported in Table 2. As can been seen from Table 2, the bifactor model showed acceptable to good fit for all reporters in both developmental periods. For the DP factor, all loadings were significant (except for the teacher reports). Some of the loadings for the scale-specific factors were also non-significant, these were mostly from the AGG scale.

**Table 2:** Fit indices for the bifactor model of DP for different reporters in middle childhood and adolescence

Developmental period	Reporter	*N*	*χ* ^2^	df	RMSEA	RMSEA 90 % CI	CFI	TLI
Middle childhood	Mother	597	1281.294	777	0.033	[0.030–0.036]	0.938	0.932
	Father	560	1147.422	777	0.029	[0.026–0.033]	0.941	0.934
	Teacher	697	3298.970	1650	0.038	[0.036–0.040]	0.928	0.923
Adolescence	Mother	479	1142.179	777	0.031	[0.027–0.035]	0.952	0.947
	Father	445	1077.051	777	0.029	[0.025–0.034]	0.950	0.944
	Teacher	419	2637.617	1650	0.038	[0.035–0.040]	0.944	0.939
	Youth	476	1323.920	777	0.038	[0.035–0.042]	0.912	0.902

### Measurement invariance across gender, parents, and time

Table 3 shows the results for the tests for measurement invariance across gender, parents, and time. All models showed good fit. ΔCFI and ΔRSMEA indicated that for both developmental periods, the restricted models across gender and across parents did not fit significantly worse. Also, the restricted model over time did not fit significantly worse. It can thus be concluded that the DP construct is invariant across gender and parents in middle childhood and in adolescence and that it is invariant across time.

**Table 3:** Measurement invariance of the bifactor model of DP across gender, parents, and time in middle childhood and adolescence using CBCL mother reports

	*χ* ^2^	df	RMSEA	RMSEA 90 % CI	CFI	TLI	Δdf	ΔCFI	ΔRMSEA
Across gender									
Middle childhood									
Model 1: less restrictive model	1910.872	1554	0.028	[0.023–0.032]	0.950	0.944			
Model 2: metric and scalar invariance	1998.153	1630	0.028	[0.023–0.032]	0.948	0.945	76	0.002	0.000
Adolescence									
Model 1: less restrictive model	1951.260	1554	0.033	[0.028–0.037]	0.942	0.936			
Model 2: metric and scalar invariance	2069.602	1630	0.034	[0.029–0.038]	0.936	0.932	76	0.006	0.001
Across parents									
Middle childhood									
Model 1: less restrictive model	4039.699	3272	0.020	[0.018–0.022]	0.943	0.939			
Model 2: metric and scalar invariance	4076.765	3348	0.019	[0.017–0.021]	0.946	0.943	76	0.003	0.001
Adolescence									
Model 1: less restrictive model	3811.037	3272	0.018	[0.016–0.021]	0.955	0.952			
Model 2: metric and scalar invariance	3858.223	3348	0.018	[0.015–0.020]	0.957	0.956	76	0.002	0.000
Across time									
Model 1: less restrictive model	3902.025	3272	0.018	[0.016–0.020]	0.954	0.951			
Model 2: metric and scalar invariance	4000.231	3348	0.018	[0.016–0.020]	0.952	0.950	76	0.002	0.000

### Relations of dysregulation with suicidal behavior

As a test for external validity, the DP bifactor model of each reporter was linked to a sum score of two youth-reported items measuring self-harm and suicidal ideation. For each reporter, correlations between the sum score for self-harm and suicidal ideation, the general Dysregulation factor, and each of the specific factors of Anxiety/Depression, Aggression, and Attention Problems were estimated, resulting in four correlations. For the youth-reported models we did not include items 18 and item 91 in the AD scale, as we used these items to measure self-harm and suicidal ideation.

All models showed good fit.^2^ For youth reports, the general Dysregulation factor showed a strong association with self-harm and suicidal ideation (*r* = 0.565, *p* < 0.001) but not with any of the specific factors of Anxiety/Depression (*r* = 0.095, *p* = 0.299), Aggression (*r* = 0.068, *p* = 0.475), and Attention Problems (*r* = −0.040, *p* = 0.722).

For mother reports, youth-reported self-harm and suicidal ideation was significantly related to the general Dysregulation factor (*r* = 0.264, *p* = 0.008) but not to any of the specific factors of Anxiety/Depression (*r* = 0.123, *p* = 0.218), Aggression (*r* = 0.116, *p* = 0.362), and Attention Problems (*r* = 0.139, *p* = 0.225).

For father reports, self-harm and suicidal ideation was also significantly related to the general Dysregulation factor (*r* = 0.194, *p* = 0.039) but not to any of the specific factors of Anxiety/Depression: *r* = 0.058, *p* = 0.631; Aggression: *r* = 0.201, *p* = 0.100; and Attention Problems: *r* = 0.180, *p* = 0.213).

Finally, for teacher reports, self-harm and suicidal ideation was not related to either the general Dysregulation factor (*r* = −0.032, *p* = 0.784), the specific Anxiety/Depression factor (*r* = −0.046, *p* = 0.707), or the specific Attention Problems factor (*r* = −0.043, *p* = 0.698). Self-harm and suicidal ideation was however significantly associated to the specific factor of Aggression (*r* = 0.288, *p* = 0.048).

In sum, youth-reported self-harm and suicidal ideation was only significantly related to the general Dysregulation factor and not to any of the specific AAA-factors when mothers, fathers, and adolescents reported on DP. For teacher reports, only specific Aggression was associated with self-harm and suicidal ideation.

## Discussion

We tested whether DP is best conceptualized as comorbidity (i.e., a second-order model) or as a syndrome, either completely (i.e., a one-factor model) or next to specific problems of anxiety/depression, aggression, and attention problems (i.e., a bifactor model). Our results provided convincing support for the hypothesized bifactor structure. This indicates that, both in middle childhood and adolescence, DP is best conceptualized as a *syndrome*, which exists over and above to specific problems of anxiety and depression, aggression, and attention problems. These results might help explain findings of specific as well as general etiology factors in the development of anxiety/depression, aggression, and attention problems [e.g., 10, 12, 24]. In addition, it might help explain the heterogeneity in symptom presentation over time.

Importantly, the DP bifactor structure was successfully replicated for father, teacher, and youth reports, both in middle childhood and adolescence. Furthermore, measurement invariance across parents was examined which demonstrated that the DP structure similarly appeared across fathers and mothers in assessing their children and adolescents. Measurement invariance across time demonstrated the equivalence of DP across two developmental periods (middle childhood and adolescence). These replications show the robustness of the bifactor DP structure across parents and time and underscore that a differentiation should be made between a general syndrome, representing DP, and the specific problems of the AAA-scales. Moreover, the results are in line with findings of Althoff et al. [3], who demonstrated that teachers, parents, and youth themselves all identified a specific subgroup of dysregulated children who showed elevated symptoms on each of the AAA-scales. As we used different statistical techniques (CFA vs LCA), our results suggest that both with person- and variable-centered approaches the structure of DP can be validated across reporters. Our results underscore the conclusion that mothers, fathers, teachers, and youth reports of child and adolescent problem behavior similarly define the factor structure of DP, suggesting that all three reporters could be used in future research and clinical practice. We could examine measurement invariance only across parents due to a different constellation as well as different number of items for parents, teachers, and youths.

Our study is the first to examine whether the conceptualization of DP is similar for boys and girls by examining whether the bifactor model was invariant across gender. The same bifactor structure of DP was found for boys and girls, both in middle childhood and adolescence. With measurement invariance across gender, means and variances of DP between boys and girls can be reliably compared in future studies.

One of the major advantages of bifactor models in the study of DP is that it can help disentangling common and unique variance of the specific factors of Anxiety/Depression, Aggression, and Attention Problems versus the general Dysregulation factor, and therefore, it is a promising model to use in further research.

We tested external validity of the bifactor model by linking the DP bifactor model for each of the reporters to adolescents’ reported self-harm and suicidal ideation. Interestingly, when youth themselves, their mothers, or their fathers were reporters, the general Dysregulation factor was found to be related to higher rates of concurrent self-harm and suicidal ideation as reported by adolescents themselves, whereas none of the specific factors of AD, AGG, and AP showed any relation to suicidality. For teacher reports, only AGG was related to self-harm and suicidal thoughts or behaviors. One explanation is that in the TRF, a relatively smaller part of the items measures symptoms of anxiety and depression, whereas a larger part measures symptoms of aggression. Furthermore, the constellation of items in subscales differs between the CBCL, TRF, and YSR, which could affect the results when linking DP to external measures. Also, these teachers only spend a few hours each week with the adolescent, thereby obtaining a different perspective on the adolescent’s behavior than his or her parents.

These findings are in line with the results of Althoff et al. [3], who studied the cross-informant agreement of DP using LCA. They reported that children in the DP class as identified by parental and youth reports had a heightened risk for suicidal thoughts and behaviors (especially when both parent and child placed the child in the DP class). However, children identified by teachers as being dysregulated did not show a heightened risk of self-reported suicidal thoughts and behaviors.

Furthermore, these results indicate that the previously demonstrated link between DP and suicidal behavior [e.g., 2, 9] was not an artifact of only elevated AD behavior causing a high DP score. Moreover, this finding is in line with LCA research showing that only the DP class showed elevated suicidal ideation [30], again indicating the uniqueness of DP as a construct next to other forms of psychopathology. These findings add to previous literature indicating that comorbidity of psychiatric problems especially is related to suicidality [44]. Replication in larger, and possibly clinical, samples is necessary. Nonetheless, the moderate to strong relations between mother-, father-, and youth-reported DP with youth-reported self-harm and suicidal thoughts and behavior in this community sample already underscores the need for study on DP as a high-risk marker for severe problems.

### Suggestions for future research

As this study used a community sample, the results have to be replicated for clinical samples in order to further validate the factor structure of DP. Another suggestion is to examine the relations between DP and other measures of self-regulation in order to further validate the construct. Furthermore, as the development of self-regulatory capacities is an important developmental task in early childhood [45], it is important to examine Dysregulation in young children as well. We have recently conducted such a study on DP using a sample of predominantly clinically referred preschoolers [46]. In this study, we aimed to replicate and further validate the bifactor structure of DP using the CBCL/1.5-5. The results showed that a bifactor model fitted the data better than a second-order and a one-factor model for both parent-reported and teacher-reported DP. In addition, analyses on criterion validity showed that the general DP factor and the specific AAA-factors were differentially related to different markers of dysregulation and clinically relevant criteria like sleep problems and inhibition.

Finally, as the DP makes use of three scales that comprise a broad range of the CBCL syndrome scales, it is likely that children scoring high on Dysregulation would also score high on the so-called p factor that has been reported in recent studies [e.g., 21, 22]. The p factor describes liability to developing psychopathology in general, and emotional dysregulation has been found to be a salient early developmental feature of the p factor [21]. Further research could elucidate the distinction and developmental timing of the DP and the p factor.

### Implications for clinical practice

Bifactor models have several implications for clinical practice (see [20]), and the bifactor DP model may therefore have great potential for clinical use. For example, bifactor models can suggest subtypes for DSM diagnoses. They can also inform treatment decisions by suggesting that treatment should be tailored to symptom profile. For example, a bifactor model of DP could suggest that treatment of anxiety/depression, aggression, and attention problems shares identical components, most likely in targeting children’s self-regulatory capacities. Symptom presentation could provide further information on how to tailor the treatment to the child’s needs.

Another implication of the bifactor structure of Dysregulation is that the use of the subscales as independent sources of information should be discouraged. High scores on a specific subscale (e.g., AGG) should be considered within the broader spectrum of psychopathology. For example, a child scoring high only on the AGG subscale of the CBCL or TRF needs a different treatment approach than a child that scores high on the AGG subscale *and* the AD subscale. Also, the results of this study suggest that when examining co-occurring behavior problems in children and adolescents, it is important to look beyond co-occurring Internalizing and Externalizing behavior problems (in the higher order structure of the CBCL) and consider Attention Problems as well. For younger children, examining co-occurring Internalizing and Externalizing Problems is thought to be similar to examining the DP [47] as the AP subscale is part of broad-band scale Externalizing Problems for the CBCL/1.5-5. However, in the CBCL/6-18, only Aggressive and Rule-breaking Behavior make up the broad-band scale Externalizing Problems, and therefore, this conclusion does not necessarily hold for older children.

The DP can help in identifying children who have problems with self-regulation across all its components (affect, behavior, and cognition). As DP can describe dysregulation problems using scores of only three scales, the profile is much more parsimonious and clearly interpretable than a Total Problems score on the CBCL. Although high Total Problems scores could also indicate dysregulation problems, high scores might also indicate a large amount of problems within the internalizing or externalizing spectrum only.

Clinicians could make use of this profile by assessing whether children show elevations on *all three* of the AAA-scales, as a way of classifying children as having dysregulation problems. One possible future direction might be to add the DP in the ASEBA [13]. As this study added to a growing body of research demonstrating that different forms of psychopathology are often underlain by more general factors [21, 22], it is recommended that clinicians always consider high scores on a specific subscale within the broader spectrum of psychopathology. For example, when a child is referred for aggressive problems it is advisable to examine as well whether a child suffers also from anxiety. Moreover, clinicians can be ascertained that DP has a similar structure in boys and girls and that mother, father, teacher, and youth reports can be used to assess this profile.

## Conclusion

In conclusion, this study adds to the existing body of research on DP as a broad syndrome of dysregulation by demonstrating that a bifactor model best represents the AAA-scales that constitute DP. This syndrome exists next to specific problems of anxiety and depression, aggression, and attention problems both in middle childhood and adolescence, and for mothers, fathers, teachers, and youth themselves as reporters. The bifactor DP model was invariant across gender, across parents, and across time and was uniquely associated to youth-reported self-harm and suicidal ideation, underscoring the severity of dysregulatory problems. With the bifactor model, general and specific factors can be teased apart, providing the opportunity to examine predictors, consequences, and the development of DP in a more refined way.

## Footnotes

1 A second-order model is statistically indistinguishable from a model with three correlated latent factors.

2 Fit statistics can be obtained from the first author
